# Functioning and activity outcomes of the Akwenda Intervention Program for children and young adults with cerebral palsy in Uganda: A cluster‐randomized trial

**DOI:** 10.1111/dmcn.16007

**Published:** 2024-06-25

**Authors:** Elizabeth Asige, Gillian Saloojee, Carin Andrews, Lukia H. Namaganda, Angelina Kakooza‐Mwesige, Diane L. Damiano, Hans Forssberg

**Affiliations:** ^1^ Department of Pediatrics and Child Health Makerere University Kampala Uganda; ^2^ Department of Physiotherapy, Faculty of Health Sciences University of the Witwatersrand Johannesburg South Africa; ^3^ Department of Women's and Children's Health Karolinska Institutet Stockholm Sweden; ^4^ SWEDESD, Department of Women's and Children's Health Uppsala University Uppsala Sweden; ^5^ Department of Epidemiology and Biostatistics Makerere University School of Public Health Kampala Uganda; ^6^ Rehabilitation Medicine Department Clinical Center, National Institutes of Health Bethesda MD USA; ^7^ Astrid Lindgren Children's Hospital Stockholm Sweden

## Abstract

**Aim:**

To evaluate the efficacy of the Akwenda Intervention Program on motor, self‐care, and social function of children and young people with cerebral palsy (CP).

**Method:**

This was a cluster‐randomized, controlled, single‐blinded, intervention study of 100 participants with CP (2–23 years; 52 males) in rural eastern Uganda. Half were allocated to the intervention program, the remainder served as waitlist controls. Gross Motor Function Measure‐66 (GMFM‐66) and the Ugandan version of Pediatric Evaluation of Disability Inventory (PEDI‐UG) were collected before group allocation and after intervention. General linear models and *t*‐tests were used to compare changes within and between groups. Cohen's *d* estimated the effect size of group differences. Change scores were evaluated by age and mobility subgroups.

**Results:**

Significant group by time interactions were found for GMFM‐66 (*p* =0.003) and PEDI‐UG outcomes (*p* <0.001), except mobility, with the intervention group demonstrating greater changes. Both groups increased their scores on the GMFM‐66 and child PEDI‐UG, while only the intervention group had significant increases in caregiver assistance scores and across all age and mobility subgroups. Cohen's *d* showed large effect sizes (*d* >0.8) of differences for PEDI‐UG outcomes except mobility.

**Interpretation:**

The Akwenda Intervention Program had a large positive impact on functioning and activity across age and mobility levels.

## Graphical abstract

Functioning and activity outcomes of the Akwenda Intervention Program for cerebral palsy in Uganda.

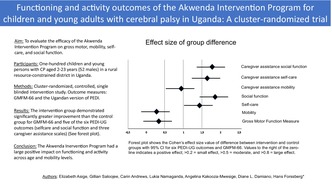

This original article is commented by Olusanya on pages 10–11 of this issue.

**Abbreviations**
GMFM‐66: Gross Motor Function Measure‐66
PEDI‐U: Pediatric Evaluation of Disability Inventory (Ugandan version)

**What this paper adds**

- The Akwenda Intervention Program dramatically improved social function and self‐care skills, and to a lesser degree gross motor function.
- The intervention had the largest effects on the need for caregivers' assistance, indicating higher levels of independence.
- The program had similar impact across ages and functional levels.

Cerebral palsy (CP) is among the most common causes of childhood‐onset disability, ranging in prevalence across geographical regions and economic conditions. While a decreasing trend exists in high‐income countries in Europe and Australia (from 2.3 per 1000 to 1.6 per 1000 in the past decade) recent studies from low‐ and middle‐income countries in sub‐Saharan Africa show nearly twice the prevalence (3.4 per 1000).^1^, ^2^, ^3^ The 2019 Global Burden of Disease study placed CP among health conditions with the highest years of lived disability because of its severity and lifelong duration.^4^ Motor disorders are the core features of CP, ranging from minimal to severe activity restrictions.^5^ Additionally, many have hearing, vision, cognition, communication, or behavioural difficulties, and seizures that exacerbate the disability. There are enormous global challenges to meet the rehabilitation needs of this large population.^4^ Children with CP living in sub‐Saharan Africa also encounter discrimination, neglect, and exclusion from health care and education services owing to limited understanding and cultural beliefs, with many families seeking help from traditional healers, religious leaders, or herbalists.^6^, ^7^, ^8^, ^9^ Rehabilitation services, assistive devices, and trained professionals are lacking, and where services are available they are often not grounded on scientific evidence.^10^, ^11^

Here, we evaluated an intervention program specifically developed for children and young people with CP living in resource‐constrained settings with limited health specialists.^12^ It was based on three key principles: (1) the framework of the International Classification of Functioning, Disability and Health; (2) evidence‐based practice; (3) universal health access; and was designed to meet challenges we had identified in studies of a population‐based cohort of children with CP in a rural region of Uganda with higher prevalence,^2^ excessive premature mortality,^13^ poor growth with more than two‐thirds being malnourished,^14^ poor development, lower school attendance, and severely restricted participation in daily activities.^10^, ^15^, ^16^ Caregivers described financial hardship in seeking care for their child, inadequate knowledge on how to help their child, and lost hope in seeing any improvement.^10^ Three intervention goals were identified: (1) improve motor function, activity, and participation of children; (2) increase knowledge and skills, and improve mental health and quality of life of caregivers; (3) reduce stigma in the community and facilitate inclusion.^12^

To achieve these goals, the Akwenda Intervention Program was developed by a team of international experts, and Ugandan academic and health professionals.^12^ When searching for evidence‐based intervention programs for improving motor function and activity for children and young people above 2 years of age from low‐ and middle‐income countries, we only found a recent community‐based parent training program in Ghana which had positive impacts on caregivers' quality of life but no effect on children's health.^17^ We therefore searched for evidence‐based interventions from high‐income countries with the aim of adapting them to low‐resource settings. A recent review summarized the increasing number of clinical trials on interventions demonstrating efficacy, or lack thereof, for improving motor function in children with CP.^18^ Features common to effective interventions were ‘practice of real‐life tasks and activities, using self‐generated active moments, at high intensity, where the practice directly targets the achievement of a goal set by the child’.^18^ We incorporated this ‘child‐active’ approach in both caregiver‐ and therapist‐led workshops and goal‐directed training, including use of technical assistive devices. Caregivers, not therapists, provided the skill training to their child and worked to achieve their preset goals.^12^

For this broad and multicomponent program, we used a series of tools measuring outcomes at the levels of the (1) child or young person, (2) caregiver, and (3) community. This is the first in a series of several planned publications evaluating the Akwenda Intervention Program and focuses on outcomes related to functioning and activity of the participant with CP, while participation, and caregiver and community outcomes, will be presented in subsequent reports. The hypothesis was that participants in the intervention group would improve more than those in the control group. We also explored whether effects differed depending on sex, age, and functional level.

## METHOD

### Study setting and design

This was a cluster‐randomized, controlled, single‐blinded study on a cohort of children and young people with CP in the Iganga‐Mayuge Health and Demographic Surveillance Site covering a population of approximately 80 000 inhabitants in 65 villages in a mainly rural area in eastern Uganda. Most inhabitants were engaged in subsistence farming and living below or close to the poverty level (for details see Andrews et al.^16^). There was one general hospital, 16 community‐based health centres with a network of village health team workers, and two non‐governmental organizations providing some services for children with disabilities.

The CP cohort was divided in two arms: one receiving the Akwenda Intervention Program, the other serving as control waiting to receive the intervention the following year. Baseline characteristics and baseline outcome measures were assessed before allocation in groups, and outcome measures were repeated after the intervention period. Children with seizures in both groups were provided with antiseizure medications. All caregivers provided written informed consent, and assent was obtained from study participants whenever possible, to the research and to publication of the results.

The study was approved by the Uganda National Council for Science and Technology (SS‐5173) and registered at the Pan‐African Clinical Trials Registry (PACTR202011738099314) and followed the published research protocol.^12^

### Participants

A total of 100 children and young people with CP aged 2 to 23 years were included (52 males). Most (*n* = 65) were from a population‐based cohort identified in a three‐stage screening at the Iganga‐Mayuge Health and Demographic Surveillance Site^2^ in 2015 which has been followed and studied in a series of articles.^10^, ^13^, ^14^, ^15^, ^16^ An additional group of 35 children between ages 2 years and 6 years were conveniently sampled to include younger participants and achieve statistical power. CP diagnosis was confirmed by a child neurologist using the definition of the Surveillance of Cerebral Palsy in Europe.^19^

All participants and their caregivers living in the same and neighbouring villages were clustered together, resulting in two geographically defined groups. The reason for this was to gather families close to the venue of the workshops for each of the four caregiver groups, and to reduce contamination of the communication and advocacy intervention. The two groups were stratified by age, sex, and Gross Motor Function Classification System (GMFCS) level. The two groups were recruited from 35 and 30 villages respectively, with a range of one or two participants from each village. A coin flip was used to decide which group should receive the intervention first. Demographic characteristics are presented in Table 1.

**TABLE 1:** Baseline characteristics of children and young people with cerebral palsy and their caregivers collected in 2021 before randomization.

Category		All (*n* = 94, 100%)	Intervention (*n* = 48, 100%)	Control (*n* = 46, 100%)	Pearson's *χ* ^2^	*p*
Age, years	2–5	17 (18)	10 (21)	7 (15)	0.605	0.739
	6–12	48 (51)	23 (48)	25 (54)		
	13–23	29 (31)	15 (31)	14 (30)		
Sex	Female	42 (45)	19 (40)	23 (50)	1.031	0.310
	Male	52 (55)	29 (60)	23 (50)		
Residence area	Semi‐urban	27 (29)	13 (27)	14 (30)	0.129	0.720
	Rural	67 (71)	35 (73)	32 (70)		
Primary caregiver	Mother	50 (53)	28 (58)	22 (48)	4.432	0.618
	Grandmother	25 (27)	10 (21)	15 (33)		
	Father	13 (14)	7 (15)	6 (13)		
	Other	6 (6)	3 (6)	3 (6)		
Primary caregiver occupation	Subsistence farmer	48 (51)	24 (50)	24 (52)	5.375	0.146
	Petty trade	33 (35)	18 (38)	15 (33)		
	Formal employed	4 (4)	3 (6)	1 (2)		
	Others	9 (10)	3 (6)	6 (13)		
GMFCS level, 2021	I	25 (27)	16 (33)	9 (20)	6.608	0.158
	II	20 (21)	10 (21)	10 (22)		
	III	9 (10)	3 (6)	6 (13)		
	IV	21 (22)	8 (17)	13 (28)		
	V	19 (20)	11 (23)	8 (17)		
Marital status	Married	64 (68)	33 (69)	31 (67)	0.802	0.670
	Separated	20 (21)	11 (23)	9 (20)		
	Widowed	10 (11)	4 (8)	6 (13)		
Level of education	Unknown	10 (11)	7 (14)	3 (7)	9.424	0.224
	None	5 (5)	1 (2)	4 (9)		
	Primary	53 (56)	24 (50)	29 (63)		
	Secondary	18 (19)	10 (21)	8 (17)		
	Tertiary/university	8 (9)	6 (13)	2 (4)		
Monthly income (Ugandan shillings)	<100 000	55 (59)	29 (60)	26 (57)	7.736	0.102
	100 000–200 000	26 (28)	14 (29)	12 (26)		
	200 000–500 000	6 (6)	5 (10)	1 (2)		
	>500 000	1 (1)	0 (0)	1 (2)		
	Unknown	6 (6)	0 (0)	6 (13)		
Age of primary caregivers, years	26–45	43 (46)	21 (44)	22 (48)	3.308	0.191
	46–65	40 (42)	22 (46)	18 (39)		
	66–85	11 (12)	5 (10)	6 (13)		
Cohort Origin	Original	63 (67)	33 (69)	30 (65)	0.133	0.716
	New recruits	31 (33)	15 (31)	16 (35)		

*Note*: Data are *n* (%) unless otherwise stated. The table only includes those participating in both the baseline and follow‐up assessments. Pearson's *χ* ^2^ test compared group differences at statistical significance *p* < 0.05. Monthly household income in Ugandan shillings; 1 US dollar = 3500 Ugandan shillings at the time of the study. Abbreviation: GMFCS, Gross Motor Function Classification System.

### The Akwenda Intervention Program

The Akwenda Intervention Program was described in detail in the preregistered research protocol,^12^ and the implementation is described in Appendix S1. It was conducted over 12 months (October 2021 to September 2022). Caregivers were divided into four geographical groups starting at the same time. The program included seven caregiver‐led workshops, 13 therapist‐led practical sessions, and four communication and advocacy for social and behavioural change sessions provided for each caregiver group at a venue most convenient for members of that group. Caregivers attended sessions twice a month, and their children accompanied them to the second session each month, which was the therapist‐led practical group session. To support participation, caregivers were given transport reimbursement and provided with breakfast, and an additional lunch on the days they attended the communication and advocacy workshop in the afternoon. The compliance was 93%, 95%, and 93% for the caregiver‐led, therapist‐led, and communication and advocacy sessions respectively. The intervention was coordinated by a physiotherapist/PhD student, and implemented by a team of four caregiver facilitators, three part‐time therapists, a social worker, and a community mobilizer. In addition, two sessions were held with 38 stakeholders as part of the communication and advocacy for behavioural and social change.

### Measurements

Sociodemographic information and clinical data including anthropometry, GMFCS levels,^20^ and baseline outcome measures were collected before group allocation. The primary outcome measure was the Gross Motor Function Measure‐66 (GMFM‐66), the criterion standard for measuring change in gross motor function in children with CP.^21^ GMFM‐66 raw scores were converted to scaled scores using the Gross Motor Ability Estimator software.^22^ To compare developmental rate in our cohort with children with CP in high‐income countries, we used tabulated references as the negative or positive deviation from the 50th centile^15^ from a longitudinal study in Canada of children aged 2 to 12 years.^23^ A secondary outcome was the Ugandan version of the Pediatric Evaluation of Disability Inventory (PEDI‐UG),^24^, ^25^, ^26^ a parent‐reported measure of the child's mobility, self‐care, and social function and need of caregiver assistance. PEDI‐UG raw scores were converted to scaled scores using a conversion table developed from typically developing children.^25^

The baseline assessments were performed by the same team of therapists and social worker that later implemented the intervention. The follow‐up assessments were performed by an independent team of two therapists and one social worker blinded to group allocation, and not informed about the aim or design of the project. The examiners were conversant with the local language (Lusoga) and had received 2 weeks of education and training followed by practical test assessments. All assessments were performed in the home setting of the participants.

### Statistical analysis

Data were analysed using Statistical Package for the Social Sciences (SPSS version 29.01.0, IBM Corp., Armonk, NY, USA). To evaluate whether the randomization process created similar groups, Pearson's *χ* ^2^ tests were performed at a statistical significance level of *p* < 0.05, to assess arm differences across all categorical variables describing participants' baseline characteristics. In addition, independent *t*‐tests were performed on mean GMFM‐66 scores (primary outcome) at baseline.

**FIGURE 1:**
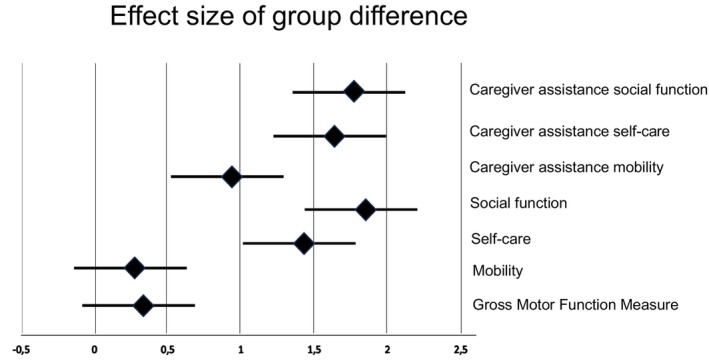
Forest plot showing the value of Cohen's *d* effect size of the group difference for each of the six Pediatric Evaluation of Disability Inventory (Ugandan version) outcomes and the Gross Motor Function Measure‐66 outcome with the 95% confidence interval for each. Values to the right of the zero‐line indicate a positive effect. The magnitude of Cohen's *d* is generally interpreted as 0.2, small effect; 0.5, moderate effect; and 0.8, large effect.

To assess whether the GMFM‐66 scores and the six PEDI‐UG scores (mobility, self‐care, and social function scaled scores and corresponding caregiver assistance scores) changed over time and by group, we first performed a standard general linear model with repeated measures of (1) pre‐ and postintervention scores as the within‐subject variable, and (2) group (intervention and control) as between‐subject variables. If significant main or interaction effects were found, post‐hoc *t*‐tests were used. We also compared the change score in each outcome measure across groups using independent *t*‐tests and the within‐group change scores using paired *t*‐tests. To estimate the magnitude of the differences across groups, effect size was computed using Cohen's *d*, calculated as the difference between the mean changes in each group over the pooled standard deviation (Table 2 and Figure 1).

**TABLE 2:** Results of the general linear mixed model presented as the *p*‐values for the GMFM‐66 and the six PEDI‐UG scales for time, main group, and group × time interaction comparing the intervention (*n* = 48) and the control (*n* = 46) groups.

Outcome measure	Main effect, time	Main effect, group	Group × time interaction	Cohen's *d*	95% CI
GMFM‐66	<0.001	0.16	0.003	0.298	−0.103 to 0.698
PEDI‐UG mobility	<0.001	0.41	0.440	0.259	−0.148 to 0.664
PEDI‐UG self‐care	<0.001	0.18	<0.001	1.444	0.983–1.898
PEDI‐UG social function	<0.001	0.70	<0.001	1.892	1.398–2.379
PEDI‐UG caregiver assistance mobility	<0.001	0.56	<0.001	0.979	0.546–1.408
PEDI‐UG caregiver assistance self‐care	<0.001	0.26	<0.001	1.642	1.168–2.110
PEDI‐UG caregiver assistance social function	<0.001	0.17	<0.001	1.756	1.273–2.232

*Note*: The effect size of the difference between groups for each outcome measure is shown using Cohen's *d* (mean and 95% CI). Abbreviations: CI, confidence interval; GMFM‐66, Gross Motor Function Measure‐66; PEDI‐UG, Pediatric Evaluation of Disability Inventory (Ugandan version).

To assess whether effects differed by age or functional level, data were subdivided in two ways: three age subgroups (2–5, 6–12, and 13–23 years) and two GMFCS subgroups (levels I and II, III–V). Change scores across groups within age or GMFCS level subgroups were compared using independent *t*‐tests. Paired *t*‐tests were used to evaluate the within‐group change for each outcome by age and GMFCS level (Tables 3, 4, 5). We also compared the magnitude of change scores across all participants by sex using independent *t*‐tests.

**TABLE 3:** GMFM‐66 scaled scores and reference scores for the intervention and control groups at baseline and follow‐up and the change, namely the difference between the two assessments.

	*n*	Intervention Baseline	Intervention Follow‐up	Intervention Change score	*p*	*n*	Control Baseline	Control Follow‐up	Control Change score	*p*	Group difference	*p*
**GMFM‐66 scale scores**												
All children	48	53.5 (27.3)	58.4 (29.2)	**4.9** (5.2)	**<0.001**	46	47.0 (24.9)	48.9 (26.6)	**1.8** (4.4)	**0.008**	**3.03**	**0.003**
Age, years												
2–5	10	28.8 (18.0)	33.1 (20.6)	**4.3** (3.8)	**0.006**	7	31.1 (12.8)	32.1 (16.0)	**1.0** (4.3)	0.580	**3.30**	0.12
6–12	23	57.4 (27.4)	62.3 (29.2)	**4.9** (5.9)	**0.001**	25	43.0 (25.1)	45.6 (26.6)	**2.5** (3.4)	**0.001**	**2.39**	0.09
13–23	15	63.9 (23.3)	69.1 (25.5)	**5.2** (5.1)	**0.002**	14	62.1 (22.4)	63.1 (25.3)	**1.0** (5.9)	0.550	**4.17**	0.052
GMFCS level												
I and II	26	75.5 (12.3)	82.2 (12.3)	**6.8** (5.7)	**<0.001**	19	72.9 (12.2)	76.6 (13.0)	**3.8** (4.8)	**0.003**	**3.02**	0.07
III, IV, and V	22	27.5 (13.7)	30.1 (13.7)	**2.6** (3.3)	**0.001**	27	28.9 (12.0)	29.3 (12.3)	**0.5** (3.6)	0.510	**2.12**	**0.04**
**GMFM‐66 reference scores**												
All children	48	−4.9 (8.6)	−0.74 (8.1)	**4.2** (5.3)	**<0.001**	46	−7.8 (8.5)	−6.1 (9.5)	**1.7** (4.4)	**0.012**	**2.51**	**0.014**
Age 2021, years												
2–5	10	−5.3 (8.2)	−3.1 (9.4)	**2.2** (3.9)	0.110	7	−7.5 (4.3)	−8.0 (7.8)	**−0.5** (4.0)	0.734	2.73	0.18
6–12	23	−5.1 (7.1)	−0.67 (6.2)	**4.4** (5.8)	**0.001**	25	−9.0 (8.2)	−6.3 (7.9)	**2.7** (3.1)	**0.001**	1.74	0.20
13–23	15	−4.4 (11.2)	0.74 (9.7)	**5.2** (5.1)	**0.002**	14	−5.9 (10.6)	−5.0 (12.7)	**1.0** (5.9)	0.545	4.17	0.052
GMFCS level 2021												
I and II	26	−4.7 (9.8)	1.2 (8.3)	**6.0** (5.9)	**<0.001**	19	−6.5 (10.5)	−3.1 (12.2)	**3.4** (4.8)	**0.007**	2.56	0.13
III, IV, and V	22	−5.1 (7.3)	−3.0 (7.3)	**2.1** (3.6)	**0.011**	27	−8.8 (6.8)	−8.3 (6.3)	**0.5** (3.7)	0.496	1.68	0.12

*Note*: Data are mean (SD). Intragroup differences between assessments were analysed by *t*‐tests and between‐group differences by comparing change scores across groups using independent *t*‐tests. Bold type indicates statistically significant values. Abbreviations: GMFCS, Gross Motor Function Classification System; GMFM‐66, Gross Motor Function Measure‐66.

## RESULTS

Of the 100 participants enrolled in the trial, 94 completed the study: 48 in the intervention group and 46 in the control group (Figure S1). In the intervention group, two participants withdrew whereas three children in the control group were deceased at follow‐up and one child withdrew. The randomization process was successful in producing groups similar in all 11 baseline characteristics (*p* > 0.05; Table 1) and in gross motor function (GMFM‐66; *p* = 0.16; Table 3). One child in the intervention group (GMFCS levels I and II; age 6–12 years) only had complete GMFM‐66 data because PEDI‐UG could not be assessed at follow‐up.

### Comparing changes between intervention and control groups

Results from the general linear models are shown in Table 2. Significant main effects for time were seen for all outcomes, indicating that functioning increased over time in both groups. Group by time interactions were found for the GMFM‐66 (*p* = 0.003) and all PEDI‐UG outcomes (*p* < 0.001) except mobility, indicating that the magnitude of increase was significantly greater in the intervention group.

When evaluating change scores, the GMFM‐66 change score (mean 4.9 [SD 5.2] vs 1.8 [SD 4.4]; *p* = 0.003; Table 3) and all PEDI‐UG change scores were significantly greater in the intervention group (*p* = 0.001; Tables 4 and 5) except for the PEDI‐UG mobility child score. The effect size of the group differences calculated by Cohen's *d* is also presented as a forest plot (Figure 1). Effect sizes for the GMFM‐66 and PEDI‐UG mobility were small (<0.5) while these were large (>0.80) for all other outcomes.

**TABLE 4:** PEDI‐UG scaled scores for the intervention and control groups at baseline and follow‐up, and the change score between the two assessments.

PEDI‐UG mobility	*n*	Intervention Baseline	Intervention Follow‐up	Intervention Change score	*p*	*n*	Control Baseline	Control Follow‐up	Control Change score	*p*	Group difference	*p*
All children	47	55.0 (24.2)	58.6 (25.2)	**3.6** (4.5)	**<0.001**	46	51.6 (22.2)	54.5 (23.6)	**2.8** (4.4)	**<0.001**	**0.78**	0.40
Age, years												
2–5	10	37.1 (13.8)	40.9 (15.9)	**3.8** (3.4)	**0.006**	7	35.9 (14.8)	38.0 (16.3)	**2.1** (5.6)	0.352	**1.68**	0.45
6–12	22	57.0 (26.6)	60.9 (27.3)	**3.9** (5.6)	**0.003**	25	49.0 (19.3)	52.0 (20.0)	**3.1** (3.6)	**<0.001**	**0.83**	0.54
13–23	15	64.2 (20.6)	67.1 (22.3)	**2.9** (3.7)	**0.008**	14	64.2 (24.6)	66.8 (27.2)	**2.6** (5.5)	0.097	**0.32**	0.86
GMFCS level												
I and II	25	75.0 (6.9)	79.3 (8.50)	**4.4** (4.7)	**<0.001**	19	74.2 (7.4)	78.3 (9.6)	**4.1** (4.8)	**0.002**	**0.30**	0.84
III, IV, and V	22	32.4 (14.8)	35.1 (14.6)	**2.7** (4.3)	**0.008**	27	35.7 (13.5)	37.6 (13.7)	**1.9** (4.0)	**0.021**	**0.78**	0.52
**PEDI‐UG self‐care**												
All children	47	52.9 (27.3)	59.6 (29.3)	**6.7** (3.6)	**<0.001**	46	48.2 (24.5)	49.4 (24.9)	**1.2** (4.0)	**0.044**	**5.52**	**<0.001**
Age 2021, years												
2–5	10	26.7 (9.1)	31.8 (10.6)	**5.1** (2.0)	**<0.001**	7	30.2 (9.5)	31.3 (9.6)	**1.1** (1.3)	0.069	**4.00**	**<0.001**
6–12	22	56.8 (26.7)	64.0 (28.9)	**7.2** (3.7)	**<0.001**	25	44.1 (23.6)	46.5 (24.5)	**2.4** (4.1)	**0.007**	**4.79**	**<0.001**
13–23	15	64.6 (25.8)	71.8 (27.7)	**7.2** (4.2)	**<0.001**	14	64.4 (22.7)	63.6 (24.1)	**−0.8** (4.1)	0.482	**7.99**	**<0.001**
GMFCS level 2021												
I and II	25	**75.6** (13.2)	**84.0** (13.8)	**8.4** (3.6)	**<0.001**	19	**73.2** (11.6)	**75.2** (12.1)	**2.0** (4.1)	0.050	**6.39**	**<0.001**
III, IV, and V	22	**27.1** (11.2)	**31.9** (12.6)	**4.9** (2.7)	**<0.001**	27	**30.5** (12.7)	**31.2** (11.9)	**0.7** (3.9)	0.370	**4.19**	**<0.001**
**PEDI‐UG social function**												
All children	47	48.7 (29.8)	57.1 (30.6)	**8.4** (3.7)	**<0.001**	46	43.9 (28.0)	45.3 (27.8)	**1.3** (3.8)	**0.023**	**7.12**	**<0.001**
Age 2021, years												
2–5	10	25.0 (15.5)	32.6 (16.0)	**7.6** (2.6)	**<0.001**	7	25.0 (11.8)	28.4 (12.1)	**3.4** (1.7)	**0.002**	**4.20**	**0.002**
6–12	22	53.6 (29.7)	62.9 (31.2)	**9.3** (3.7)	**<0.001**	25	42.4 (27.0)	44.2 (27.4)	**1.9** (3.5)	**0.014**	**7.46**	**<0.001**
13–23	15	57.3 (30.3)	65.0 (30.3)	**7.7** (4.3)	**<0.001**	14	56.3 (30.6)	55.5 (31.0)	**−0.7** (4.2)	0.530	**8.40**	**<0.001**
GMFCS level 2021												
I and II	25	72.7 (16.7)	82.0 (16.5)	**9.3** (4.1)	**<0.001**	19	71.6 (19.1)	72.6 (19.7)	**0.9** (4.1)	0.344	**8.37**	**<0.001**
III, IV, and V	22	21.4 (12.5)	28.9 (13.0)	**7.4** (3.1)	**<0.001**	27	24.5 (12.0)	26.0 (12.0)	**1.6** (3.6)	**0.029**	**5.86**	**<0.001**

*Note*: Data are mean (SD). Intragroup differences between assessments were analysed by *t*‐tests and between‐group differences by comparing change scores across groups using independent *t*‐tests. Bold type indicates statistically significant values. Abbreviations: GMFCS, Gross Motor Function Classification System; PEDI‐UG, Pediatric Evaluation of Disability Inventory (Ugandan version).

**TABLE 5:** PEDI‐UG caregiver's assistance scaled scores for the intervention and control groups at baseline and follow‐up, and the change score between the two assessments.

PEDI‐UG Mobility	*n*	Intervention Baseline	Intervention Follow‐up	Intervention Change score	*p*	*n*	Control Baseline	Control Follow‐up	Control Change score	*p*	Group difference	*p*
All children	47	38.3 (29.5)	43.7 (30.2)	**5.5** (4.8)	**<0.001**	46	37.5 (28.0)	37.6 (28.9)	**0.1** (6.0)	0.898	**5.35**	**<0.001**
Age, years												
2–5	10	13.9 (20.0)	19.0 (22.4)	**5.1** (6.0)	**0.025**	7	16.4 (13.9)	19.9 (16.5)	**3.5** (7.9)	0.280	**1.57**	0.65
6–12	22	40.3 (29.7)	46.3 (30.2)	**6.0** (5.3)	**<0.001**	25	35.0 (28.2)	33.7 (28.2)	**−1.3** (5.7)	0.276	**7.25**	**<0.001**
13–23	15	51.5 (25.5)	56.5 (26.3)	**5.0** (3.3)	**<0.001**	14	52.3 (26.0)	53.2 (28.9)	**0.9** (5.2)	0.530	**4.08**	**0.02**
GMFCS level												
I and II	25	63.9 (6.3)	70.0 (3.4)	**6.2** (4.0)	**<0.001**	19	68.1 (4.1)	68.6 (5.1)	**0.5** (5.3)	0.680	**5.65**	**<0.001**
III, IV, and V	22	9.2 (13.7)	13.9 (15.2)	**4.7** (5.6)	**<0.001**	27	15.9 (13.3)	15.7 (15.1)	**− 0.2** (6.6)	0.901	**4.85**	**0.01**
**PEDI‐UG self‐care**												
All children	47	50.7 (37.1)	59.0 (39.2)	**8.4** (5.6)	**<0.001**	46	46.6 (33.3)	47.0 (32.9)	**0.4** (3.8)	0.431	**7.91**	**<0.001**
Age 2021, years												
2–5	10	14.1 (18.2)	20.4 (20.9)	**6.4** (5.4)	**0.005**	7	21.1 (15.4)	21.3 (17.2)	**0.1** (4.2)	0.938	**6.23**	**0.02**
6–12	22	56.4 (34.8)	66.0 (37.8)	**9.6** (6.5)	**<0.001**	25	42.4 (32.7)	43.4 (31.6)	**0.9** (4.4)	0.292	**8.61**	**<0.001**
13–23	15	66.7 (34.7)	74.6 (35.1)	**7.9** (4.3)	**<0.001**	14	66.7 (30.4)	66.4 (31.2)	**−0.3** (2.3)	0.642	**8.20**	**<0.001**
GMFCS level 2021												
I and II	25	82.0 (12.8)	92.4 (12.2)	**10.4** (5.3)	**<0.001**	19	80.5 (16.2)	80.0 (16.5)	**−0.4** (3.0)	0.551	**10.83**	**<0.001**
III, IV, and V	22	15.1 (18.3)	21.1 (19.3)	**6.0** (5.2)	**<0.001**	27	22.7 (17.4)	23.8 (18.0)	**1.0** (4.2)	0.206	**4.96**	**<0.001**
**PEDI‐UG social function**												
All children	47	45.6 (35.2)	57.0 (36.4)	**11.3** (5.5)	**<0.001**	46	41.9 (34.8)	41.5 (33.4)	**−0.4** (7.7)	0.737	**11.74**	**<0.001**
Age 2021, years												
2–5	10	21.4 (28.3)	32.0 (29.0)	**10.6** (5.5)	**<0.001**	7	19.3 (11.7)	18.6 (14.4)	**−0.7** (7.6)	0.818	**11.30**	**0.003**
6–12	22	48.3 (35.2)	60.0 (37.5)	**11.7** (6.2)	**<0.001**	25	40.6 (34.7)	42.0 (32.4)	**1.4** (8.4)	0.417	**10.30**	**<0.001**
13–23	15	57.8 (33.4)	69.2 (32.8)	**11.4** (4.7)	**<0.001**	14	55.6 (37.6)	52.1 (37.8)	**−3.4** (5.6)	**0.040**	**14.77**	**<0.001**
GMFCS level 2021												
I and II	25	74.6 (15.5)	86.6 (15.7)	**11.8** (5.8)	**<0.001**	19	73.1 (27.5)	71.8 (24.6)	**−1.3** (9.3)	0.537	**13.32**	**<0.001**
III, IV, and V	22	12.7 (17.6)	23.3 (19.7)	**10.7** (5.2)	**<0.001**	27	19.9 (18.9)	20.2 (19.4)	**0.3** (6.5)	0.816	**10.36**	**<0.001**

*Note*: Data are mean (SD). Intragroup differences between assessments were analysed by *t*‐tests and between‐group differences by comparing change scores across groups using independent *t*‐tests. Bold type indicates statistically significant values. Abbreviations: GMFCS, Gross Motor Function Classification System; PEDI‐UG, Pediatric Evaluation of Disability Inventory (Ugandan version).

### Analysis by sex, and age and GMFCS level subgroups

There was no difference in change scores because of sex.

Within‐groups paired *t*‐tests showed that all age and GMFCS subgroups in the intervention group had significant increases in GMFM‐66 despite small numbers in each subgroup (Table 3). In the control group, only the middle age group (6–12 years) and the GMFCS levels I and II subgroup showed significant improvements. When comparing GMFM change scores across groups using independent *t*‐tests, those in GMFCS levels III to V subgroup had greater change in the intervention group (*p* = 0.006) with a similar trend in the oldest age subgroup (*p* = 0.052). The results of the GMFM reference scores, taking into account expected development by age, were similar with significantly greater changes in the intervention group as a whole compared with the control group (*p* = 0.014), and the same trend in the oldest age subgroup (*p* = 0.052).

There was a significant increase over time for all PEDI‐UG outcomes in all intervention subgroups (Tables 4 and 5), while the change was significant only in some of the control subgroups. There were no significant positive changes in any of the PEDI‐UG caregiver assistance scores for the control group (Table 5). Instead, the only significant change was a decrease in social function in the oldest age group. PEDI‐UG self‐care and social function child scores and all caregiver assistance scores showed greater changes in all age and GMFCS subgroups (except for the youngest age subgroup for caregiver assistance mobility).

## DISCUSSION

Results demonstrated conclusively that the Akwenda Intervention Program had a broad and large impact on daily functioning. The intervention group had significantly larger changes than the control group in almost all outcome measures. The greatest impact was on social function and self‐care skills, while being smaller on gross motor function and mobility. The large impact on PEDI‐UG caregiver assistance scales suggests that participants needed less assistance and became more independent. The intervention was effective in all age and functional level groups, with no sex difference.

To explore the effectiveness of the multicomponent Akwenda Intervention Program, we followed the guidance for complex interventions in the real world developed to evaluate the impact of interventions with several interconnected components designed to affect multiple outcomes through several pathways,^27^ complementing general guidelines on how to design, implement, and report randomized controlled trials.^28^ We used a randomized controlled trial design because it supports inference about causal relationships and assigned participants to study arms before intervention, making the direction of causality clear. We randomized clusters of participants and demonstrated that we had two equivalent arms for a range of baseline characteristics (*n* = 11). We used an intention‐to‐treat design including participants even if they did not fully adhere to the intervention protocol. The compliance was very high among caregivers (93%), and the attrition was small: only two participants withdrew in the intervention arm and one in the control group. Three children died in the control group which may or may not have been related to group assignment. However, since we had not included mortality as an outcome measure in our pre‐trial research protocol, this is only an observation. We adhered to the three principles in evaluating complex interventions.^27^ First, the outcome measures were selected by the goals of the intervention and linked to clearly stated hypothesizes, namely to improve gross motor function (GMFM‐66), mobility, self‐care skills, and social function (PEDI‐UG), which were the registered outcomes. Second, the trial was adequately powered. Third, we registered the research protocol including design and outcome measures in advance (PACTR202011738099314),^12^ and adhered to the protocol without any changes. Strictly following all these procedures, finding statistically significant differences with large effect sizes between the intervention and control arms, we have demonstrated the effectiveness of this real‐world complex intervention, and that the intervention improved gross motor function, self‐care skills, and social function.

The group analysis demonstrated significantly greater changes in the intervention versus control group for GMFM‐66 and nearly all PEDI‐UG outcomes. Effect size is particularly useful to illustrate group differences (see Figure 1) which ranged from small positive effects on the GMFM‐66 to very large effect sizes (>1.4) for PEDI‐UG self‐care and social function. Only the intervention group improved in caregiver assistance scores, resulting in very large effect sizes (>1.6) for self‐care and social function, and large effect sizes (>0.8) for PEDI‐UG mobility, reflecting participants achieving greater independence. When comparing GMFM‐66 scores with tabulated reference centiles,^23^ both groups had negative reference scores at baseline, reflecting non‐optimal development probably as a result of lacking interventions. The negative reference score was dramatically reduced in the intervention group, indicating that the intervention was effective in promoting a larger increase than the expected natural development. The minimum clinical important difference has been developed for GMFM‐66 to determine whether an improvement is clinically meaningful.^29^ In the intervention group, 87% of participants in GMFCS levels I to III had large meaningful changes. The impact on gross motor function was remarkable considering that all child‐directed therapy was performed by family members with minimal individualized one‐to‐one therapy by professionals. The results agree with a recent report on a community‐based, family‐centred intervention program in Bangladesh provided by caregivers and locally trained therapists showing improved GMFM‐66 scores in children younger than 5 years.^30^

The outcome profile, with the largest effects on self‐care skills, social function, and caregivers' assistance scales, corresponds with the intervention focus to educate caregivers about CP and coach them on how to care for and motivate their child. It is not possible to identify which program components contributed to specific outcomes but the change of the caregivers' attitudes towards their children probably played an essential role. Caregivers realized that their children could do more for themselves and thus expected more from them. It also fostered greater inclusion in family and other social activities instead of being neglected owing to lack of knowledge and stigma. Likewise, provision of technical assistive devices would primarily affect independence and inclusion rather than improving motor skills. The goal‐setting component incorporating daily training towards goals also probably contributed to improved child functioning, as shown in a study from the Netherlands.^31^ Overall, the child‐functioning improvement by the Akwenda Intervention Program stands out in a global context compared with studies from high‐income countries. A systematic review of training in task‐specific gross motor skills identified eight randomized controlled trials with positive within‐group effects but conflicting between‐group effects for skill performance and functional skills, and no group difference or negative effects for gross motor function.^32^ The large improvements shown in the evaluation of the Akwenda Intervention Program were probably possible because participants had no previous therapy, reflected by large negative GMFM reference scores, while participants in studies of high‐income countries had received previous interventions.

The panorama of the CP cohort was very broad, with ages ranging from 2 years to 23 years, and GMFCS ranging from levels I to V. We questioned whether the intervention was more effective for specific ages or functional levels. Despite small samples, all the intervention subgroups improved in almost all outcome measures, with significant group differences in change scores in five PEDI‐UG outcomes, two exceptions being in PEDI‐UG mobility and GMFM. Thus, it is apparent that the Akwenda Intervention Program is effective for those with mild and severe functional limitations, as well as for small children to young adults.

On the basis of a previous 4‐year follow‐up study of partly the same cohort, we expected the control group would exhibit no increase in GMFM‐66 and PEDI‐UG mobility scores, and only small increases in PEDI social and self‐care scores.^15^ Surprisingly, they showed significant increases in both GMFM and many PEDI‐UG scores. Several factors could have contributed to the increase. Being in a study alone can lead to positive effects reflected by within‐group improvements of most control groups in other CP intervention studies.^31^, ^32^ The provision of antiseizure medications to both groups might have influenced development in the control participants. There could also have been ‘contamination’ from the intervention, influencing children in the control group. We tried to reduce this by clustered assignment of neighbouring villages, but the communication and advocacy program probably spread to villages and families of the control group. Two non‐governmental organizations giving care to children with disability had also started recently, and some children in the control group had visited these clinics. There might also have been bias introduced by having two different teams administering assessments before and after the intervention. The new ‘blinded’ assessors were trained by the original team to calibrate the assessments, but interrater reliability was not assessed. Thus, it is possible that the second assessment team gave systematically higher scores.

To our knowledge, this is one of the first reported randomized controlled intervention studies aimed at improving outcomes for children and young people with CP living in a rural, resource‐constrained setting in sub‐Saharan Africa. The study's strengths include the rigorous adherence to methodological criteria for complex interventions in the real world,^27^ including randomized, intention‐to‐treat design, two equivalent arms, low attrition and high compliance, preregistration of the trial, and outcome measures selected by the goals of the intervention and blinded postintervention assessments on a population‐based cohort many of whom had been followed for 4 years. However, the study setting presented challenges. The high premature mortality reduced the numbers of participants requiring recruitment of new children and the cohort was no longer strictly population‐based. We assigned participants in geographical clusters because the workshops were performed in four groups of caregivers living close to the venue. We also wanted to minimize contamination between arms, for example from the communication and advocacy activities. The assessors performing the follow‐up assessments were blinded, were not informed about the randomized controlled trial design of the project, and had no invested interest in the outcome. Yet, assessors could have noticed clues such as geography or technical assistive devices that could have compromised the blinding. The unexpected improvement of the control group exposed other limitations, such as contamination bias, potential influence of antiseizure medication and other service providers, and absence of interrater reliability between the first and second teams of assessors. This last factor would, however, have affected both groups similarly.

## CONCLUSION

The Akwenda Intervention Program promoted large and meaningful improvements in social functions, self‐care skills, and gross motor function for children and young people with CP, resulting in children becoming less dependent on their caregivers. Rehabilitation professionals are limited resources in most low‐ and middle‐income countries, and the concept and design of the Akwenda Intervention Program, including trained parent facilitators, is thus an effective approach to maximizing the reach of therapists. Hopefully, the dramatic improvements in children's functioning in this study will inspire global change to group‐based, family‐centred, and parent‐led interventions.

## FUNDING INFORMATION

Swedish Research Council, Stiftelsen Frimurare Barnhuset i Stockholm, Stiftelsen Sunnerdahls Handikappfond, and Stiftelsen Promobilia.

## CONFLICT OF INTEREST STATEMENT

The authors have declared that they had no interests that might be perceived as posing a conflict or bias.

## Supporting information


**Appendix S1:** Implementation of the Akwenda Intervention Program.

**Figure S1:** Flow chart of participants.

## Data Availability

The data that underlie the results reported in this article are described at the Swedish National Data Service. Dataare made available upon request after ensuring compliance with relevant legislation.https://doi.org/10.48723/k9wd‐9j90 .
