# Supplementary material for: Functioning and activity outcomes of the Akwenda Intervention Program for children and young adults with cerebral palsy in Uganda: A cluster‐randomized trial
Source: Dev Med Child Neurol. 2024 Jun 25;67(1):87–98. doi: 10.1111/dmcn.16007 (PMC11625466; doi:10.1111/dmcn.16007)
Supplement: Supplementary file 2 — Figure S1: Flow chart of participants. [file DMCN-67-87-s001.pptx]

## Slide 1
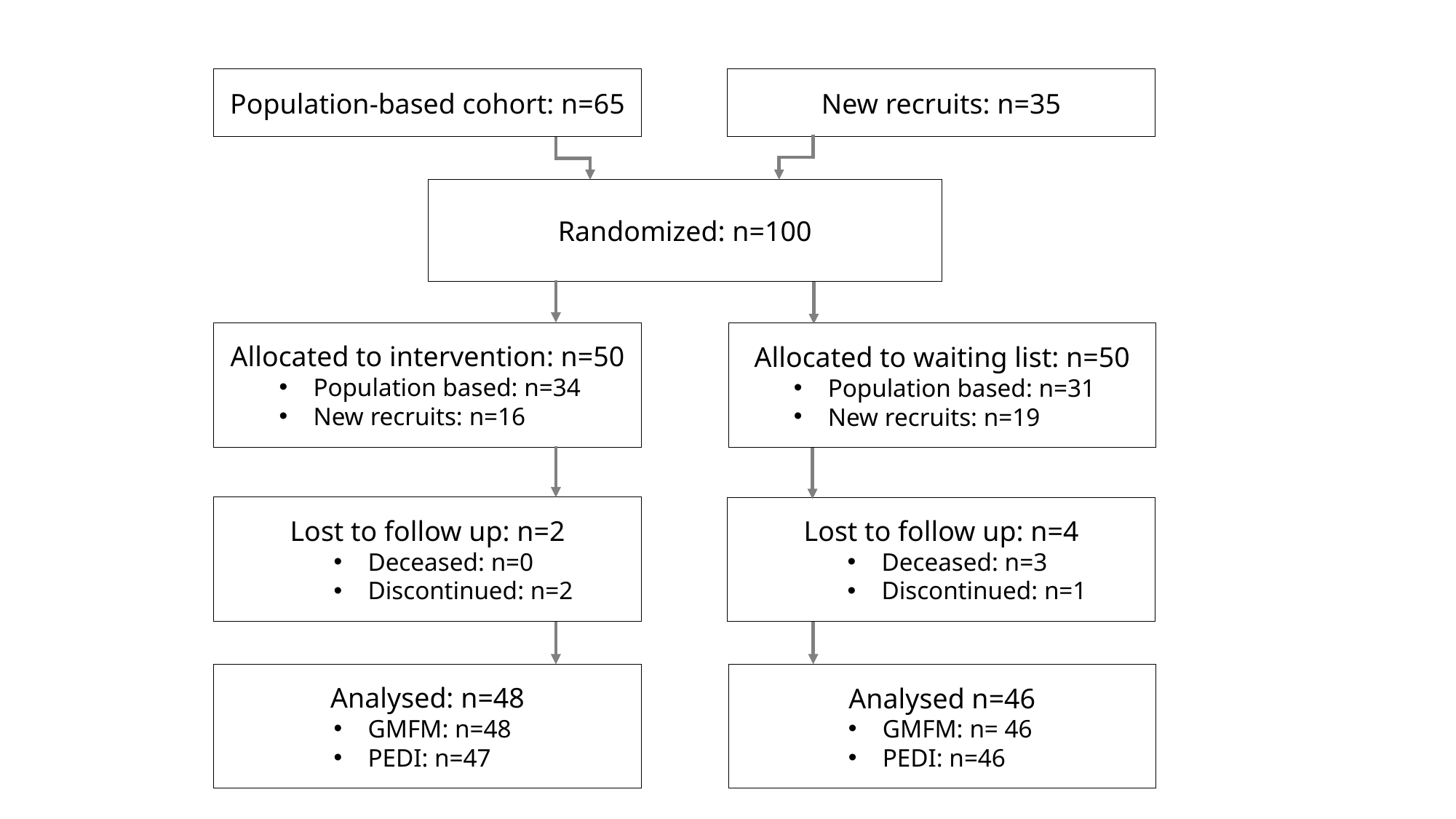

New recruits: n=35
Population-based cohort: n=65
Randomized: n=100
Allocated to intervention: n=50
Population based: n=34
New recruits: n=16
Allocated to waiting list: n=50
Population based: n=31
New recruits: n=19
Lost to follow up: n=4
Deceased: n=3
Discontinued: n=1
Lost to follow up: n=2
Deceased: n=0
Discontinued: n=2
Analysed: n=48
GMFM: n=48
PEDI: n=47
Analysed n=46
GMFM: n= 46
PEDI: n=46
